# Interpreting Mixture Profiles: Comparison Between Precision ID GlobalFiler™ NGS STR Panel v2 and Traditional Methods

**DOI:** 10.3390/genes11060591

**Published:** 2020-05-26

**Authors:** Michele Ragazzo, Stefania Carboni, Valerio Caputo, Carlotta Buttini, Laura Manzo, Valeria Errichiello, Giulio Puleri, Emiliano Giardina

**Affiliations:** 1 Department of Biomedicine and Prevention, Tor Vergata University of Rome, 00133 Rome, Italy; michele.ragazzo@uniroma2.it (M.R.); v.caputo91@gmail.com (V.C.); carlotta.buttini@students.uniroma2.eu (C.B.); laura.manzo@uniroma2.it (L.M.); valeria.errichiello@uniroma2.it (V.E.); giuliopuleri@gmail.com (G.P.); 2 Genomic Medicine Laboratory UILDM, Santa Lucia Foundation IRCCS, 00142 Rome, Italy; stefaniacarboni58@gmail.com

**Keywords:** forensic genomics, DNA typing, next-generation sequencing (NGS), mixtures

## Abstract

Forensic investigation for the identification of offenders, recognition of human remains, and verification of family relationships requires the analysis of particular types of highly informative DNA markers, which have high discriminatory power and are efficient for typing degraded samples. These markers, called STRs (Short Tandem Repeats), can be amplified by multiplex-PCR (Polymerase Chain Reaction) allowing attainment of a unique profile through which it is possible to distinguish one individual from another with a high statistical significance. The rapid and progressive evolution of analytical techniques and the advent of Next-Generation Sequencing (NGS) have completely revolutionized the DNA sequencing approach. This technology, widely used today in the diagnostic field, has the advantage of being able to process several samples in parallel, producing a huge volume of data in a short time. At this time, although default parameters of interpretation software are available, there is no general agreement on the interpretation rules of forensic data produced via NGS technology. Here we report a pilot study aimed for a comparison between NGS (Precision ID GlobalFiler™ NGS STR Panel v2, Thermo Fisher Scientific, Waltham, MA, USA) and traditional methods in their ability to identify major and minor contributors in DNA mixtures from saliva and urine samples. A quantity of six mixed samples were prepared for both saliva and urine samples from donors. A total of 12 mixtures were obtained in the ratios of 1:2; 1:4; 1:6; 1:8; 1:10; and 1:20 between minor and major contributors. Although the number of analyzed mixtures is limited, our results confirm that NGS technology offers a huge range of additional information on samples, but cannot ensure a higher sensitivity in respect to traditional methods. Finally, the Precision ID GlobalFiler™ NGS STR Panel v2 is a powerful method for kinship analyses and typing reference samples, but its use in biological evidence should be carefully considered on the basis of the characteristics of the evidence.

## 1. Introduction

Massively Parallel Sequencing (MPS), also known as Next-Generation Sequencing (NGS) is highly useful to analyze high-throughput DNA sequencing for biotechnology discovery purposes, including genes associated with specific diseases or a group of related disorders or forensic DNA typing [1,2,3,4,5].

The application of NGS technology in the field of forensic genetics may provide additional information when compared to traditional approaches such as Short Tandem Repeats (STRs) analysis and Capillary Electrophoresis (CE). The high throughput NGS analysis allows the generation and detection of millions of short sequencing reads in a single machine run [6]. This approach may facilitate the detection of STRs, SNPs (Single Nucleotide Polymorphisms) and indels (insertion and deletion) simultaneously [7]. In this regard, the possibility of studying very short DNA fragments offers advantages in the analysis of degraded DNA increasing the discrimination power of the method [8,9]. In addition, NGS, discriminating the isometric alleles, can significantly improve the discrimination power of routinely used markers [8]. Forensic NGS is suitable for human identification, phenotyping, ancestry and mixture interpretation [8,10,11]. 

To date, several forensic panels have been developed and in particular, we studied the "Precision ID GlobalFiler ™ NGS STR Panel v2 with the Ion S5™ system (Thermo Fisher Scientific, Waltham, MA, USA) that is composed by 31 autosomal STRs:

- 20 expanded Combined DNA Index System (CODIS) core loci: TPOX, D3S1358, FGA, D5S818, CSF1PO, D7S820, D8S1179, TH01, vWA, D13S317, D16S539, D18S51, D21S11, D1S1656, D2S1338, D2S441, D10S1248, D12S391, D19S433 and D22S1045;
- 11 non-CODIS loci: D1S1677, D2S1776, D3S4529, D4S2408, D5S2800, D6S1043, D6S474, D12ATA63, D14S1434, Penta E and Penta D;
- 4 gender determination loci: Amelogenin, DYS391, SRY and Y-indel (rs2032678) [2].

Here we report a pilot study aimed to compare NGS (Precision ID GlobalFiler™ NGS STR Panel v2) and traditional methods in their ability to identify major and minor contributors in DNA mixtures from saliva and urine samples. A quantity of six mixed samples were prepared for both saliva and urine samples from donors. A total of 12 mixtures were obtained in the ratios of 1:2, 1:4, 1:6, 1:8, 1:10, and 1:20 between minor and major contributors. 

Subsequent biostatistical analysis was used for the evaluation of the weight of the evidence (presence or absence of minor and major contributors in the profile) through the ratio between prosecution Hypothesis (Hp) and defense Hypothesis (Hd). As usual, we used the Likelihood Ratio (LR) approach to compare the relationship between two mutually exclusive hypotheses (presence/absence) for each contributor. 

## 2. Materials and Methods 

### 2.1. Selection of Sample

Ten unrelated individuals (6 females and 4 males) were recruited in order to test traditional and NGS technologies [2]. All subjects provided 1 buccal swab and 1 urine sample, for a total of 10 buccal swabs and 10 urine samples marked with a specific internal code, as shown in Supplementary Table S1. Informed consent was obtained from each volunteer before the sample collection [12,13,14]. The study was approved by the Ethics Committee of Santa Lucia Foundation (CE/PROG.650 approved on 01/03/2018).

Two male subjects were selected for the mixture analysis. In particular, 6 mixed samples were prepared starting from the buccal swabs and 6 mixed samples were obtained from urine. A total of 12 mixtures were collected in the ratios of 1:2, 1:4, 1:6, 1:8, 1:10, and 1:20, as reported in Supplementary Table S2.

### 2.2. Purification and Quantification

The samples were processed applying the standard laboratory protocol in order to perform the comparative analysis. The Genomic DNA extraction required a manual lysis phase followed by automated extraction through “Maxwell® 16 MDx Instrument” (Promega, WI, USA) in association with the kit “DNA IQ™ Casework Pro Kit for Maxwell® 16” (Promega, WI, USA) according to the manufacturer’s instruction. Quantifiler® Trio DNA Quantification Kit (Thermo Fisher Scientific, Waltham, MA, USA) was used to evaluate the concentration and the quality of the purified DNA followed by analysis through the software “HID Real-Time PCR Analysis Software” according to the instructions provided by the kit. The Quantifiler™ Trio DNA Quantification Kit simultaneously assesses DNA concentration and degradation by detecting two human multicopy autosomal targets: a small amplicon of 80 bp in length and a large amplicon of 214 bp size. Using the ratio between the concentrations of both amplicons (short/large) the level of degradation could be measured. Thus, the larger target is more likely to be affected by degradation of the DNA template than the shorter autosomal target. When DNA is degraded, the concentration of DNA detected with the degradation target is less than the concentration detected with the autosomal target.

### 2.3. Traditional Method

Capillary Electrophoresis (CE) is the most common method for the examination of STRs loci used in forensic genetics workflow. The procedure requires the analysis of 20 or more different loci amplified in a single reaction thorough PCR multiplex [15]. In our study, the quantified samples were amplified and characterized by PCR-multiplex using the GlobalFiler™ PCR Amplification Kit (Thermo Fisher Scientific, Waltham, MA, USA) following manufacturer’s protocols [16]. The separation and detection of the amplicons were conducted byApplied Biosystems® 3130xl Genetic Analyzer (Thermo Fisher Scientific, Waltham, MA, USA) according to manufacturer’s protocols [17]. The collection and the analysis of data by CE were assessed by GeneMapper™ ID-X Software v1.5 (Applied Biosystems).

### 2.4. NGS Method

The extracted DNA was sequenced using Ion S5™ System (Ion Torrent™) (ThermoFisher Scientific, Foster City, CA, USA) [3]. 

Library preparation was performed automatically using the Precision ID GlobalFiler ™ NGS STR Panel v2 with Ion AmpliSeq™ technology. This system types 35 STRs markers, including 20 CODIS STRs, 9 multiallelic STR markers, 4 useful markers for the determination of sex and two Penta-STRs (Penta D and Penta E) with high informativity. The DNA input quantity was normalized to achieve 1ng in 15 μL, for an input concentration of 0.067 ng/μL. For this study, the construction of the automated library has been conducted using the Ion Chef™ instrument through IonAmp Seq^TM^ Chef Reagents DL8 and IonAmp Seq^TM^ Chef Solution DL8. Four different runs of the Ion Chef were performed (8 samples each). After that, target libraries were quantified in duplicate using the Ion Library TaqMan® Quantitation Kit on QuantStudio™ 5 Real-Time PCR System according to the manufacturer’s instructions. The positive control employed in this work is the *Escherichia coli* DH10B Control Library. This control was used with the serial diluted concentrations detected in duplicate (6.8 pM, 0.68 pM, 0.068 pM and the negative control of nuclease-free water) [2]. The reaction mix included 10 μL of Ion Library qPCR Master Mix, 1 μL of Ion Library TaqMan® Quantitation Assay 20X, 9 μL of the 1:100 diluted sample library or dilution control library or negative control for a total reaction volume per well of 20 μL. The thermal cycling conditions consisted of a polymerase activation at 95 °C for 1 min, followed by 29 cycles of 94 °C for 10 s, and 59 °C for 90 s and a final incubation at 60 °C for 10 min.

After the automated library preparation, the four libraries (8 pooled samples each) were quantified and diluted to a concentration of 50 pM and subsequently combined in two library pools (template). The library was prepared using a volume of 25 uL of each library pool loaded on Ion Chef System. Samples were run on Ion S5 instrument using two Ion 520TM Chip (650 flows). After about 10 h the IonChips were loaded into the Ion S5 Sequencer ™ (Thermo Fisher Scientific, Waltham, MA, USA) and sequenced using the Ion S5 Precision ID Sequencing Kit. The data were extracted from the S5 Torrent Server v5.10.0 (Thermo Fisher Scientific, Waltham, MA, USA) and subsequently analyzed using the HID Genotyper plugin-2.1 (Thermo Fisher Scientific, Waltham, MA, USA) according to default parameters, after alignment to the Homo Sapiens hg19 reference genome.

Converge software v2.1 (Thermo Fisher Scientific, Waltham, MA, USA) was then used to interpret STR results, as indicated in the user’s manual. The Depth of Coverage (DoC) was calculated as the number of unique reads specific for each locus; the Sequence Coverage Ratios (SCRs), including the Allele Ratio (AR), stutter ratio and noise ratio, were determined as the relative proportion of sequences for alleles, stutter and noise [2].

The two methods shared the extraction of DNA and quantification analysis. Subsequently, the DNA was tested using traditional assays (GlobalFiler® PCR amplification Kit, Thermo Fisher Scientific, Waltham, MA, USA) and NGS method (Precision ID GlobalFiler ™ NGS STR Panel v2, Thermo Fisher Scientific, Waltham, MA, USA). The GlobalFiler® PCR amplification Kit allows the simultaneous analyses of 21 autosomic loci-STR, 3 Y chromosome loci-STR, an indel marker located on Y chromosome and the amelogenin locus for sex determination. It is to notworthy that the Precision ID GlobalFiler ™ NGS STR Panel v2 analyzes a higher number of markers (35), including 20 CODIS STRs, 9 multiallelic STRs, 4 markers for the determination of sex and two penta-STR markers with high informativity [18]. These two markers can be useful during interpretation of mixed profiles or complex paternity testing.

The comparative analysis between the two methods was performed by evaluating only the shared markers between the two methods (Supplementary Table S3). 

The NGS analysis was performed using the HID Genotyper plugin v2.1 (Thermo Fisher Scientific, Waltham, MA, USA) with default thresholds. In particular, the relative analytical and stochastic thresholds were both 0.05 (the thresholds of the software, for every marker, was 5% of the total coverage of the alleles in the locus), whereas the CE thresholds were calculated experimentally through the internal validation of the accredited internal method [2,19,20]. In particular, the values applied were 45 RFU (Relative Fluorescence Unit) for the Analytical Threshold (AT) and 277 RFU for the Stochastic Threshold (ST).

### 2.5. Statistical Approach and Semi-Continuous Method

According to the international guidelines, statistical analysis required the evaluation of the weight of the evidence (presence or absence of individuals in the profile) through the ratio between prosecution Hypothesis (Hp) and defense Hypothesis (Hd). These hypotheses are utilized for the evaluation of the strength of evidence provided by genetic analysis [21]. The Likelihood Ratio (LR) is the relationship between two mutually exclusive hypotheses. In particular, the Hp is considered as the accusatory hypothesis for which a known profile (such as the profile of the suspect) is a contributor to the mixed profile, and the Hd as the defensive hypothesis for which the known profile is not a contributor to the mixed profile. When the LR is greater than 1, the evidence favors Hp; when it is less than 1, the evidence favors Hd.

To evaluate the probabilities of all possible genotype groups in the mixture profiles and to calculate the LR for a series of propositions, we applied a widely used software for a semi-continuous model (LRmix Studio) [21]. This “discrete” model requires the presence or absence of peaks along with probabilities of allele drop-out or drop-in and its implementation always needs specialized software [1,21]. Therefore, the LRmix Studio software is a probabilistic genotyping software (PGS) to assist DNA mixture interpretation [1,22,23,24,25].

### 2.6. Quality Control

As usual, positive and negative controls were run demonstrating the absence of contamination and the specificity of the reactions.

The traditional method of this research was performed in the Tor Vergata Forensic Genetics Laboratory of Tor Vergata University of Rome accredited to the norm UNI CEI EN ISO/IEC 17025:2018 for the tests recorded in the official list, which is available on ACCREDIA website [26]. 

NGS experiments are performed using the Synthetic Library (Test Fragments), which provides information on the experiment performance. This approach allowed to estimate the carry-forward (CF)/ incomplete extension (IE)/ droop (DR) values and monitor the system characteristic. Test Fragment is characterized by a short-known sequence which is recognized by the system in order to monitor the correct amplification of the libraries. A JSON-format file is the format required from the system to analyse the Test Fragments.

## 3. Results

### 3.1. Single DNA Profiles

The results of quantification and degradation were reported in the Supplementary Table S4 and S5. The quality data were reported as ratio between small autosomal target DNA conc. (ng/μL) and Large autosomal target DNA conc. (ng/μL). This value is automatically calculated by the HID Real-Time PCR Software.

The percentage of usable reads was 44%. Alignment parameters showed good values, confirming the reliability of the results. In particular, out of a total of 180 M aligned reads, the total number of bases with an alignment error rate of 2% or less (AQ17) was 141 M, while 125 M of bases had an error rate of alignment equal to 1% (AQ20). All recruited samples were sequenced using two chips obtaining 1,928,901 and 1,824,130 reads respectively. The ratios of the aligned read were high and ranging from 99.6% to 99.8%.

Based on these results, metrics such as DoC and SCR were calculated and compared. These showed similar results across sample sources. In particular, the DNA obtained from buccal swabs revealed that the average DoC across the expected 23 loci was 44.38× ± 16.28× (mean ± SD), while the average DoC obtained from urine samples was 43.12× ± 20.61× (mean ± SD) (Table 1).

**Figure 1:**
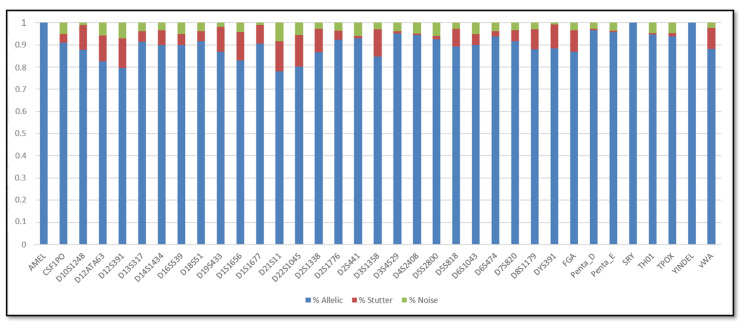
Sequence coverage ratios of the buccal swab samples.

**Figure 2:**
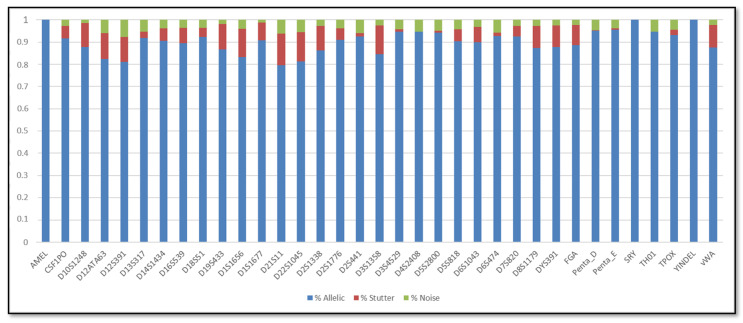
Sequence coverage ratios of the urine samples.

**Figure 3:**
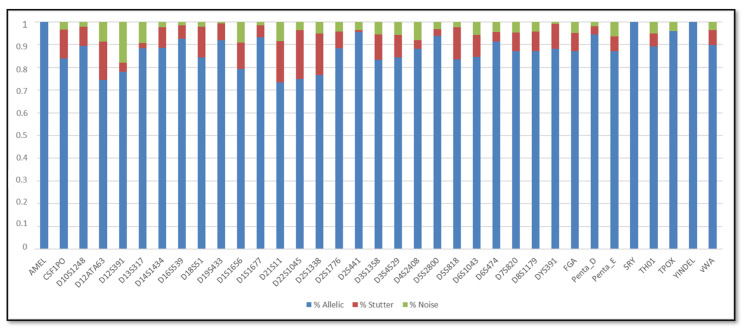
Sequence coverage ratios of the buccal swab mixtures.

**Figure 4:**
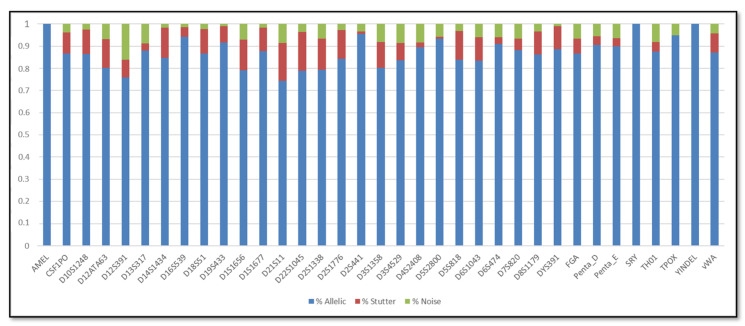
Sequence coverage ratios of the urine mixtures.

In addition, the SCRs are shown in Figure 1, Figure 2, Figure 3 and Figure 4; these include the information of SRY, Y-indel and Amelogenin, which are not STR markers and have no stutter reads, the values obtained are 1 for the allelic specific and 0 for the stutter and noise ratios.

By aligning these readings with the “in-silico” genome reference constituted by the common alleles for each locus, the NGS method correctly typed the 23 loci expected for each sample, as confirmed by the comparison with CE method. A summary of the NGS and traditional method-based genotyping results for all samples is shown in Supplementary Tables S6–S9. These results confirmed the accuracy of both the NGS and CE methods [27].

The analysis of NGS profiles, performed by default filters, revealed sequence artefacts for each locus analyzed. These artefacts, in terms of coverage and number of alleles, were dissimilar in different loci but similar between the two DNA sources. In fact, the DNA profiles of buccal swab samples reported an average coverage of the artefacts ranging between 10× at D19S433 locus and 132.25× at D16S539 locus, while the DNA profiles of the urine samples showed an average coverage of the read artefacts within the range of 10× at FGA to 128.33× at D5S818 [2].

### 3.2. Mixed DNA Profiles

The profiles obtained from 12 mixed samples were analyzed in order to compare the sensitivity of the traditional and NGS methods, with or without default filtering, according to the national and international guidelines [28,29,30]. The DNA concentration for mixture samples was reported in the Supplementary Table S10. The genotyping results for mixture samples are shown in Table 2, Table 3, Table 4 and Table 5.

**Figure 5:**
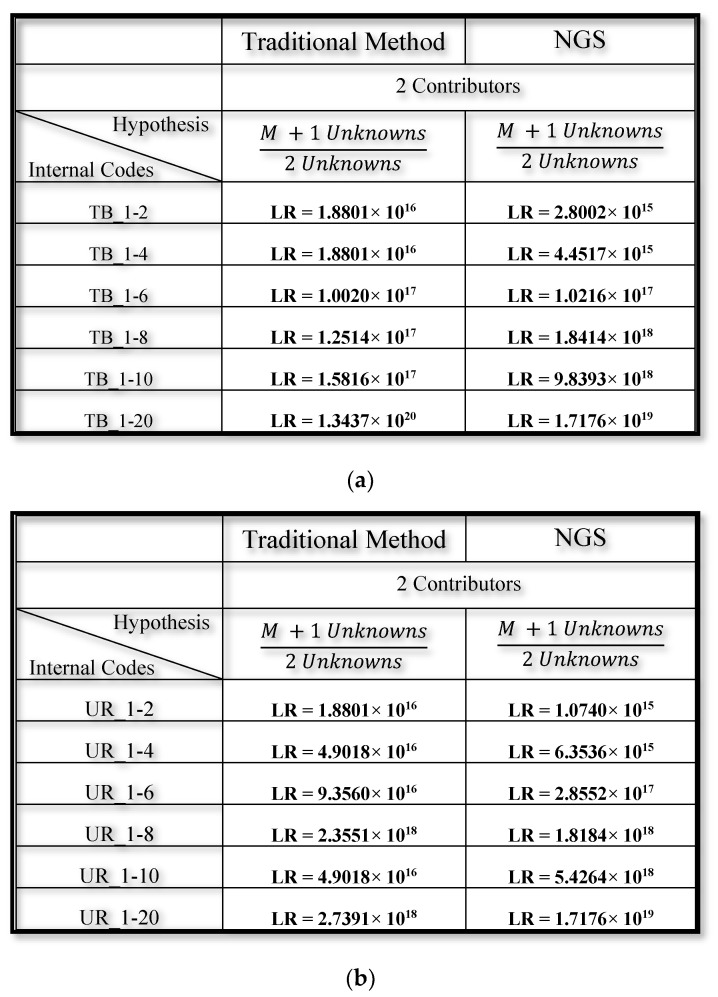
(**a**) Biostatistical and comparative analysis aimed at evaluating the inclusion or exclusion of the major contributor (M) in the mixtures coming from buccal swab sample (semicontinuous method); (**b**) biostatistical and comparative analyses aimed at evaluating the inclusion or exclusion of the major contributor (M) in the mixtures coming from urine samples (semicontinuous method).

**Figure 6:**
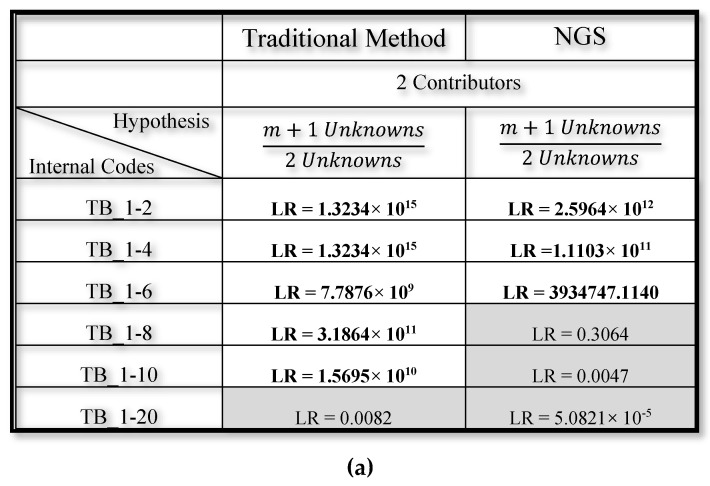

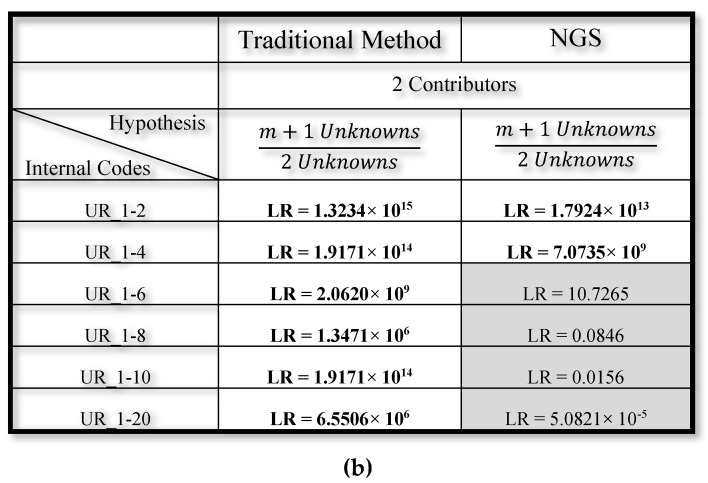
(**a**) Biostatistical and comparative analysis aimed at evaluating the inclusion or exclusion of the minor contributor (m) in the mixtures coming from buccal swab samples (semicontinuous method); (**b**) biostatistical and comparative analyses aimed at evaluating the inclusion or exclusion of the minor contributor (m) in the mixtures coming from urine samples (semicontinuous method).

The NGS biostatistical approach was performed using default filters. Afterwards, we evaluated the LR using the LRMix studio as previously mentioned and we considered the presence of major and minor contributor in a total of 2 unknown contributors. As shown in Figure 5, LR values more than 10,000, indicated by bold type, support the numerator hypothesis (the inclusion of major contributor). In Figure 6, LR values more than 10,000, indicated by hold type support the numerator hypothesis (the inclusion of minor contributor); LR less than one or neutral, indicated by gray colour, do not support the numerator hypothesis (the inclusion of minor contributor is not confirmed).

As expected, the presence of major contributors was detected using both methods for each dilution in both saliva and urine samples, demonstrating that NGS is a reliable and robust method for human identification, starting from a 1 ng amount of DNA. Finally, Capillary Electrophoresis detected the presence of the minor contributor in a 1:10 dilution for buccal swabs and in a 1:20 dilution in urine samples, whereas NGS technology only detected the presence of the minor contributor in up to 1:6 dilutions for buccal swabs and up to 1:4 dilutions in urine samples (Figure 6).

These results are a direct consequence of the application of the default analytical threshold set at 5% of the total coverage of alleles in each locus. This threshold, by definition, cannot detect minor contribution alleles when the ratio is under 1:6. 

In addition, informative variants (SNPs) within the flanking regions of the repeat motif and isometric alleles (with same length but different sequences) were analyzed in buccal swabs and urine samples [2]. SNPs located on flanking regions were: rs9546005 flanking region of D13S317, rs4847015 flanking region of D1S1656, rs25768 flanking region of D5S818, rs11642858 flanking region of D16S539 and rs75219269 flanking region of vWA. In particular, rs9546005, rs4847015 and rs25768 were detected in all tested samples. The rs11642858 and rs75219269 were detected only in major contributor and up to 1:20 dilution. Moreover, NGS analysis revealed the presence of an isometric allele 29 at D21S11 and an isometric allele 18 at D5S2800. The isometric allele 29 at D21S11 was identified in both buccal swabs and urine samples. In particular, the isometric allele 29 of major contributor ([TCTA]5 [TCTG]6 [TCTA]3 TA [TCTA]3 TCA [TCTA]2 TCCATA [TCTA]10) was detected up to 1:20 dilution, whereas the isometric allele 29 of minor contributor ([TCTA]4 [TCTG]6 [TCTA]3 TA [TCTA]3 TCA [TCTA]2 TCCATA [TCTA]11) was detected up to 1:10 dilution (the system did not distinguish real allele from stutter peak). The isometric allele 18 ([GGTA]3 [GACA]10 [GATA]2 [GATT]3) was detected in the minor contributor, up to 1:6 dilutions in the buccal swab samples and up to 1:4 in urine samples. 

## 4. Discussion

In this work we report a pilot study aimed at the comparison between NGS (Precision ID GlobalFiler™ NGS STR Panel v2, Thermo Fisher Scientific, Waltham, MA, USA) and traditional methods in their ability to identify major and minor contributors in DNA mixtures from saliva and urine samples. A quantity of six mixed samples were prepared for both saliva and urine samples from donors. A total of 12 mixtures were obtained in the ratios of 1:2, 1:4, 1:6, 1:8, 1:10, and 1:20 between minor and major contributors. The analyses were conducted on 23 loci, including 20 CODIS autosomal loci STR, an STR located on the Y chromosome, an insertion/deletion marker on the Y chromosome and the amelogenin locus [31]. To our knowledge, the present study represents the first attempt to evaluate the urine as a proper DNA source for forensic purposes through NGS. We selected urine samples because they can provide a source of naturally degraded DNA [32].

Concerning the NGS quality results, the average DoCs of urine and buccal swabs DNA samples showed a substantial concordance, indicating that the different sources of the DNA do not affect the sensitivity of NGS (Table 1). Indeed, the average DNA concentration of urine samples was minor respect to the average DNA concentration of buccal swab samples (3.80 ± 4.56 ng/μL vs. 48.81 ± 25.56 ng/μL). As usual the DNA input for each sample was normalized before performing the PCR. The average DoC across the 21 (23−2) loci showed differences for buccal swab and urine samples. In particular, we observed the DoC 16× (SD) and 75× (SD) for the buccal swab samples, whereas, for urine samples, a range of 18× (SD) and 106× (SD). These extremes were found at DYS391 and TH01 for both DNA sources, respectively.

Regarding the SCR, the AR of urine DNA samples were similar to buccal swabs DNA samples. In particular, the average AR of the buccal swab mixtures was 86.5%, with the lowest (73.34%) observed for D21S11 and the highest (96%) observed for TPOX, whereas the average AR of the urine mixtures was 86.16%, with the lowest (74.34%) observed for D21S11 and the highest (95.49%) observed for D2S441. In terms of stutter, the average stutter ratio of the buccal swab mixtures and of the urine mixtures was 8.92% and 8.72% respectively; the average noise ratio was 4.86% for the buccal swab mixtures and 5.35% for the urine mixtures. All loci have shown reliability and reproducibility base on the genotyping results of the major contributor in all dilutions. 

The full concordance between the single profiles from both urine and buccal swabs, analyzed by means of NGS and CE, showed the high robustness of NGS, that can be used for the personal identification.

In forensic genetics, mixed DNA samples arise from the combination of two or more individual body fluids or secretions. Mixtures are common, and even expected, in many forensic investigations (e.g., sexual crimes, large disasters, as well as in products of conception and fingernail cuttings taken by police or at autopsy) [1,2]. DNA mixtures are often difficult to interpret because of different types of materials, number of donors and different proportions of each component in the mixture and by artifacts such as allelic dropout and allelic drop-in [1,2,33].

Regarding the mixture profiles, the goal of this analysis was to verify if the presence of major and minor contributors in the mixed samples could be stated through NGS, according to the national and international guidelines [28,29,30].

As expected, the presence of major contributors was detected using the both methods for each dilution in both saliva and urine samples, demonstrating that NGS is a reliable and robust method for human identification starting from optimal/suboptimal amount of DNA. In addition, we assessed the inclusion or exclusion of the major contributor (M) in the biological mixture. Using the 2 contributors hypothesis system, it was always possible to include the subject, both with the traditional method and the NGS method with default filters. The values were fallen into a range defined "inconclusive" when the filters were removed, confirming the importance of the pre-established analytical threshold in order to obtain useful results for comparative purposes. The relative analytical and stochastic default thresholds were set to 0.05 using the HID Genotyper plugin v2.1 (Thermo Fisher Scientific, Thermo Fisher Scientific, Waltham, MA, USA) and the CE thresholds were obtained from internal validation which led to the accreditation of the laboratory (AT = 45 RFU and ST = 277 RFU).

Different results, comparing traditional and NGS methods, were obtained through the semi-continuous approach involving two contributors, in which the inclusion or exclusion of the minor contributor (m) in biological mixtures is evaluated [34].

For example, LR calculated for sample TB_1-2 showed the highest values. It is important to underline that LR value, obtained by NGS method, results numerically lower by a factor of 3. Increasing the dilution ratio between the major contributor (M) and the minor contributor (m), the discrepancy of the values through the two methods became more marked. In fact, the TB_1-8 sample showed an LR of 3.1864 × 10^11^ through traditional methods and an inconclusive LR through NGS method with default parameters (not supporting any hypotheses).

The analyses of the urine samples, which are not considered an ideal source of DNA due to the low concentration of nucleated cells present in human urine and the instability of DNA in urine during preservation, confirmed the results obtained with buccal swab sample [32,35,36]. The dilution TB_1-8 sample, the presence of the minority contributor was detected by a value of LR = 1.3471× 10^6^, with the traditional method, while the result was inconclusive through NGS method.

The UR_1:20 sample represents the crucial point of this study underlining the extreme sensitivity of the traditional method in the study of mixed profiles. The GeneMapper software has generated an electropherogram where it is possible to highlight the presence of the minor contributor, which was not detected by the NGS method. The LR calculation confirmed these results, as shown in Figure 6. In fact, the LR value (through traditional methods) still strongly supported the accusatory hypothesis while, the LR (through NGS) strongly supported the exclusion hypothesis.

Removing the default filter, we observed different data interpretation due to the high number of detected signals, which the instrument is unable to process. In this case, the discrimination of real allelic calls and artifacts, having very high coverage values, was really challenging. As expected, different results could be observed applying different analytical threshold for NGS analysis that appear mandatory. 

Therefore, we confirm that Capillary Electrophoresis represents an extremely reliable and sensitive system, capable of detecting the presence of the minor contributor up to 1:10 dilution from buccal swab and up to dilution 1:20 in urine samples. In contrast, NGS technology detected the presence in the minor contributor only up to the 1:6 dilution starting from the buccal swabs, and only up to the 1:4 dilutions in the urine samples. 

On the other hand, the NGS method, due to the sequence-based results, can detect many signals otherwise not identifiable. The use of the “Precision ID GlobalFiler ™ NGS STR Panel v2” kit provides greater identification power, as 35 markers are analyzed against the 24 of the GlobalFiler® Amplification Kit, of the traditional method. In addition, for the interpretation of mixtures, isometric alleles (with same length but different sequences) and informative variants (SNPs) within the flanking regions of the repeat motif were used to discriminate the two contributors. [2,37]. It is interesting to notice that that two polymorphisms not shared between the two contributors (i.e., informative SNPs) were detected in all dilutions, confirming that single base variations are a sensitive marker. Unfortunately, all SNPs detected in minor contributors were shared also by the major. [37].

Setting laboratory-specific thresholds appears mandatory to ensure the correct interpretation of data and to improve the sensibility. NGS technologies are crucial for DNA human typing in cases like mass disasters or other events where forensic specimens could be reasonably attributed to a single source. 

## 5. Conclusions

Capillary Electrophoresis detected the presence of the minor contributor in 1:10 dilution for buccal swabs and in 1:20 dilution in urine samples. Conversely NGS technology detected the presence of the minor contributor only in 1:6 dilution for buccal swabs and only in 1:4 dilution in urine samples. However, the NGS method has a high power of identification because it analyzes 35 markers against 24 markers of the traditional method. In addition, Precision ID GlobalFiler ™ NGS STR Panel v2 could also detect and sequence simultaneously SNPs present in the flanking regions, providing important additional information useful for identification purposes, such as isometric alleles (with the same length but different sequences).

Furthermore, our analysis demonstrated that, in the NGS analysis, the use the default analytical threshold parameters of the software, set at 5% of the total coverage of the allele in the locus, is mandatory. When this filter was removed the false allele calls always led to misleading interpretations. Therefore, the 5% threshold allows to detect the presence of the minor contributor only in 1:6 dilution for buccal swabs and only in 1:4 dilution in urine samples. Setting laboratory-specific thresholds appears mandatory to ensure the correct interpretation of data and to improve the sensibility. Although the number of analyzed mixtures is limited, our results confirm that NGS technology offers a huge range of additional information on samples but cannot ensure a higher sensitivity respect to traditional methods. Moreover, the Precision ID GlobalFiler™ NGS STR Panel v2 is a powerful method for kinship analyses and typing reference samples, but its use in biological evidence should be carefully considered on the basis of the characteristics of the evidence.

## Figures and Tables

**Table 1:** Overview of the average DoC for each of the 23 loci regarding buccal swab and urine samples. DoC: Depth of Coverage.

Locus	Average DoC Buccal Swab	Average DoC Urine Sample
D3S1358	38.7	28.7
VWA	48.6	40.5
D16S539	64.1	63.1
CSF1PO	68.5	46.5
TPOX	66.2	66.8
AMEL	/	/
D8S1179	32.8	28.7
D21S11	52.5	37.9
DYS391	16.3	18.3
D2S441	53.7	55.0
D19S433	24.1	22.8
TH01	75.9	106.4
FGA	29.0	20.7
D22S1045	46.6	55.4
D5S818	36.2	53.9
D13S317	52.0	57.2
D7S820	51.2	42.2
D10S1248	25.6	26.8
D1S1656	34.1	31.0
D12S391	49.4	48.7
D2S1338	23.8	25.8
YINDEL	/	/
D18S51	42.8	29.3

**Table 2:** Genotyping results for the buccal swab mixture samples. CE: Capillary Electrophoresis.

CE Global Filer Mixture DNA Profiles (Buccal Swabs)							
	Sample	TB_1-2	TB_1-4	TB_1-6	TB_1-8	TB_1-10	TB_1-20
Locus							
D3S1358		14-16-17-18	14-16-17-18	14-16-17	14-16-17-18	14-16-17	14-17
VWA		14-17-18	14-17-18	14-17-18	14-17-18	14-17-18	14-17-18
D16S539		9-10-11	9-10-11	9-10-11	10-11	9-10-11	10-11
CSF1PO		10-12	10-*12*	10-12	10-12	10-12	10
TPOX		8	8	8	8	8	8
AMEL		X-Y	X-Y	X-Y	X-Y	X-Y	X-Y
D8S1179		13-16	13-16	13-16	13-16	13-16	13-16
D21S11		28-29-30	28-29-30	28-29-30	28-29-30	28-29-30	28-29-30
DYS391		10	10	10	10	10	10
D2S441		10-11	10-11	10-11	10-11	10-11	10-11
D19S433		13-14	13-14	13-14	13-14	13-14	13-14
TH01		6-9	6-9	6-9	6-9	6-9	6
FGA		20-22-23	20-22-23	20-22-23	20-22-23	20-22-23	20-22
D22S1045		15-17	15-17	15-17	15-17	15-17	15-17
D5S818		9-10-13	9-10-13	9-10-13	9-10-13	9-10-13	10-13
D13S317		8-11-13	8-11-13	8-11	8-11-13	8-11	8-11
D7S820		9-11-12	9-11-12	9-11-12	9-11-12	9-11-12	9-11
D10S1248		13-14-15	13-14-15	13-14-15	13-14-15	13-14-15	13-14-15
D1S1656		12-15-17.3	12-15-17.3	15-17.3	12-15-17.3	12-15-17.3	15-17.3
D12S391		17-17.3-22-23	17-17.3-22-23	17-17.3-22	17-17.3-22-23	17-17.3-22	17.3-22
D2S1338		17-21-25	17-21-25	17-21-25	17-21-25	17-21-25	17-25
YINDEL		2	2	2	2	2	2
D18S51		12-13	13	12-13	13	13	13

**Table 3:** Genotyping results for the buccal swab mixture samples. NGS: Next-Generation Sequencing.

NGS Global Filer Mixture DNA Profiles (Buccal Swabs)							
	Sample	TB_1-2	TB_1-4	TB_1-6	TB_1-8	TB_1-10	TB_1-20
Locus							
D3S1358		14-16-17-18	14-16-17-18	14-16-17-18	14-16-17-18	14-16-17-18	14-16-17-18
VWA		14-17-18	14-17-18	14-17-18	14-17-18	14-17-18	14-17-18
D16S539		9-10-11	9-10-11	9-10-11	9-10-11	9-10-11	9-10-11
CSF1PO		10-12	10-*12*	10-12	10-12	10-12	10
TPOX		8	8	8	8	8	8
AMEL		X-Y	X-Y	X-Y	X-Y	X-Y	X-Y
D8S1179		13-16	13-16	13-16	13-16	13-16	13-16
D21S11		28-29-30	28-29-30	28-29-30	28-29-30	28-29-30	28-29-30
DYS391		10	10	10	10	10	10
D2S441		10-11	10-11	10-11	10-11	10-11	10-11
D19S433		13-14	13-14	13-14	13-14	13-14	13-14
TH01		6-9	6-9	6-9	6-9	6-9	6-9
FGA		20-22-23	20-22-23	20-22-23	20-22-23	20-22-23	20-22
D22S1045		15-17	15-17	15-17	15-17	15-17	15-17
D5S818		9-10-13	9-10-13	9-10-13	9-10-13	9-10-13	10-13
D13S317		8-11-13	8-11-13	8-11-13	8-11-13	8-11-13	8-11-13
D7S820		9-11-12	9-11-12	9-11-12	9-11-12	9-11-12	9-11-12
D10S1248		13-14-15	13-14-15	13-14-15	13-14-15	13-14-15	13-14-15
D1S1656		12-15-17.3	12-15-17.3	12-15-17.3	12-15-17.3	12-15-17.3	12-15-17.3
D12S391		17-17.3-22-23	17-17.3-22-23	17-17.3-22-23	17-17.3-22-23	17-17.3-22-23	17-17.3-22-23
D2S1338		17-21-25	17-21-25	17-21-25	17-25	17-21-25	17-25
YINDEL		2	2	2	2	2	2
D18S51		12-13	12-13	12-13	12-13	12-13	12-13

**Table 4:** Genotyping results for the urine-mixture samples. CE: Capillary Electrophoresis.

CE Global Filer Mixture DNA Profiles (Urine)							
	Sample	UR_1-2	UR_1-4	UR_1-6	UR_1-8	UR_1-10	UR_1-20
Locus							
D3S1358		14-16-17-18	14-16-17-18	14-16-17-18	14-16-17-18	14-16-17-18	14-17
VWA		14-17-18	14-17-18	14-17-18	14-17-18	14-17-18	14-17-18
D16S539		9-10-11	9-10-11	10-11	9-10-11	9-10-11	10-11
CSF1PO		10-12	10-*12*	10-12	10	10-12	10
TPOX		8	8	8	8	8	8
AMEL		X-Y	X-Y	X-Y	X-Y	X-Y	X-Y
D8S1179		13-16	13-16	13-16	13-16	13-16	13-16
D21S11		28-29-30	28-29-30	28-29-30	29-30	28-29-30	28-29-30
DYS391		10	10	10	10	10	10
D2S441		10-11	10-11	10-11	10-11	10-11	10-11
D19S433		13-14	13-14	13-14	13-14	13-14	13-14
TH01		6-9	6-9	6-9	6-9	6-9	6-9
FGA		20-22-23	20-22-23	20-22-23	20-22-23	20-22-23	20-22
D22S1045		15-17	15-17	15-17	15-17	15-17	15-17
D5S818		9-10-13	9-10-13	10-13	10-13	9-10-13	9-10-13
D13S317		8-11-13	8-11-13	8-11-13	8-11-13	8-11-13	8-11-13
D7S820		9-11-12	9-11-12	9-11-12	9-11-12	9-11-12	9-11-12
D10S1248		13-14-15	13-14-15	13-14-15	13-14	13-14-15	13-14-15
D1S1656		12-15-17.3	12-15-17.3	12-15-17.3	12-15-17.3	12-15-17.3	12-15-17.3
D12S391		17-17.3-22-23	17-17.3-22-23	17-17.3-22	17.3-22	17-17.3-22-23	17.3-22-23
D2S1338		17-21-25	17-21-25	17-21-25	17-21-25	17-21-25	17-21-25
YINDEL		2	2	2	2	2	2
D18S51		12-13	13	12-13	13	13	13

**Table 5:** Genotyping results for the urine-mixture samples. NGS: Next-Generation Sequencing.

NGS Global Filer Mixture DNA Profiles (Urine)							
	Sample	UR_1-2	UR_1-4	UR_1-6	UR_1-8	UR_1-10	UR_1-20
Locus							
D3S1358		14-16-17-18	14-16-17-18	14-16-17-18	14-16-17-18	14-16-17-18	14-16-17-18
VWA		14-17-18	14-17-18	14-17-18	14-17-18	14-17-18	14-17-18
D16S539		9-10-11	9-10-11	9-10-11	9-10-11	9-10-11	9-10-11
CSF1PO		10-12	10-*12*	10-12	10-12	10-12	10-12
TPOX		8	8	8	8	8	8
AMEL		X-Y	X-Y	X-Y	X-Y	X-Y	X-Y
D8S1179		13-16	13-16	13-16	13-16	13-16	13-16
D21S11		28-29-30	28-29-30	28-29-30	28-29-30	28-29-30	28-29-30
DYS391		10	10	10	10	10	10
D2S441		10-11	10-11	10-11	10-11	10-11	10-11
D19S433		13-14	13-14	13-14	13-14	13-14	13-14
TH01		6-9	6-9	6-9	6-9	6-9	6-9
FGA		20-22-23	20-22-23	20-22	20-22-23	20-22-23	20-22
D22S1045		15-17	15-17	15-17	15-17	15-17	15-17
D5S818		9-10-13	9-10-13	9-10-13	9-10-13	9-10-13	9-10-13
D13S317		8-11-13	8-11-13	8-11-13	8-11-13	8-11-13	8-11-13
D7S820		9-11-12	9-11-12	9-11-12	9-11-12	9-11-12	9-11-12
D10S1248		13-14-15	13-14-15	13-14-15	13-14-15	13-14-15	13-14-15
D1S1656		12-15-17.3	12-15-17.3	12-15-17.3	12-15-17.3	12-15-17.3	12-15-17.3
D12S391		17-17.3-22-23	17-17.3-22-23	17-17.3-22-23	17-17.3-22-23	17-17.3-22-23	17-17.3-22-23
D2S1338		17-21-25	17-21-25	17-21-25	17-21-25	17-21-25	17-21-25
YINDEL		2	2	2	2	2	2
D18S51		12-13	12-13	12-13	12-13	12-13	12-13

## Supplementary Materials

The following are available online at https://www.mdpi.com/2073-4425/11/6/591/s1, Table S1: Selected samples from 10 unrelated individuals, Table S2: Different mixtures utilized in this studio, Table S3: Loci evaluated for the comparison of the two methods, Table S4: Quantification and degradation data of buccal swab samples, Table S5 Quantification and degradation data of urine samples, Table S6: Genotyping results for the 23 loci by mean of CE methodology regarding samples from buccal swabs. CE: Capillary Electrophoresis, Table S7: Genotyping results for the 23 loci by mean of NGS methodology regarding samples from buccal swabs. NGS: Next Generation Sequencing, Table S8: Genotyping results for the 23 loci by mean of CE methodology regarding samples from urine. CE: Capillary Electrophoresis, Table S9: Genotyping results for the 23 loci by mean of NGS methodology regarding samples from urine. NGS: Next Generation Sequencing, Table S10: The mixture samples DNA concentration.
