# Supplementary material for: Interpreting Mixture Profiles: Comparison Between Precision ID GlobalFiler™ NGS STR Panel v2 and Traditional Methods
Source: Genes (Basel). 2020 May 26;11(6):591. doi: 10.3390/genes11060591 (PMC7349666; doi:10.3390/genes11060591)
Supplement: Supplementary file 1 [file genes-11-00591-s001.pdf]

Table S1. Selected samples from 10 unrelated individuals.

Individuals	Sample Code Buccal Swab	Sample Code Urine
1	19-131	19-145
2	19-133	19-147
3	19-134	19-148
4	19-135	19-149
5	18-431	19-154
6	19-205	19-208
7	19-206	19-209
8	19-207	19-210
9	19-142	19-211
10	19-141	19-212

Table S2. Different mixtures utilized in this studio

Sample Code Buccal Swab	Sample Code Urine
TB_1:2	UR_1:2
TB_1:4	UR_1:4
TB_1:6	UR_1:6
TB_1:8	UR_1:8
TB_1:10	UR_1:10
TB_1:20	UR_1:20

Table S3. Loci evaluated for the comparison of the two methods.

Locus	Repeat	Chromosome location
D3S1358	TCTA/TCTG	3p21.31
vWA	TCTA/TCTG	12p13.31
D16S539	GATA	16q24.1
CSF1PO	AGAT	5q33.3-34
TPOX	AATG	2p23-2per
AMEL-X	Indel	p22.1-22.3
AMEL-Y	Indel	p11.2
D8S1179	TCTA/TCTG	8q24.13
D21S11	TCTA/TCTG	21q11.2-q21
DYS391	TCTA	Yq11.21
D2S441	TCTA/TCAA	2p14
D19S433	AAGG/TAG G	19q12
FGA	CTTT/TTCC	4q28
D22S1045	ATT	22q12.3
D5S818	AGAT	5q21-31
D13S317	TATC	13q22-31
D7S820	GATA	7q11.21-22
D10S1248	GGAA	10q26.3 8
D1S1656	TAGA	1q42.2 9
D12S391	AGAT/AGA C	12p13.2 1
D2S1338	TGCC/TTCC	2q35
Y indel	Indel	Yq11.221
D18S51	AGAA	18q21.33
TH01	TCAT	11p15.5

Table S4. Quantification and degradation data of buccal swab samples.

Individuals	Sample Code Buccal Swab	[] DNA buccal swab	Degradation Index
1	19-131	22,987 ng/μL	0,86
2	19-133	55,911 ng/μL	1,07
3	19-134	27,068 ng/μL	0,75
4	19-135	29,68 ng/μL	0,68
5	18-431	34,322 ng/μL	1,22
6	19-205	108,248 ng/μL	1,00
7	19-206	52,903 ng/μL	0,93
8	19-207	34,755 ng/μL	0,77
9	19-142	55,384 ng/μL	0,69
10	19-141	66,827 ng/μL	0,77

Table. S5. Quantification and degradation data of urine samples.

Individuals	Sample Code Urine	[] DNA Urine	Degradation Index
1	19-145	3,523 ng/μL	1,23
2	19-147	13,26 ng/μL	0,78
3	19-148	2,814 ng/μL	0,92
4	19-149	9,447 ng/μL	1,11
5	19-154	0,136 ng/μL	1,02
6	19-208	0,687 ng/μL	1,03
7	19-209	0,479 ng/μL	0,90
8	19-210	6,683 ng/μL	1,33
9	19-211	0,195 ng/μL	0,83
10	19-212	0,794 ng/μL	0,87

Table S6. Genotyping results for the 23 loci by mean of CE methodology regarding samples from buccal swabs. CE: Capillary Electrophoresis.

CE Global Filer Single DNA Profiles (Buccal Swabs)										
Sample / Locus	19-135	19-141	19-207	19-205	19-142	19-206	19-131	19-133	19-134	18-431
D3S1358	18-18	18-18	16-16	16-18	15-17	14-16	14-15	14-17	16-18	15-18
VWA	15-18	16-17	14-17	17-17	14-17	14-17	16-17	14-18	16-18	18-18
D16S539	13-13	9-11	9-13	9-11	11-12	9-12	12-13	10-11	11-12	9-11
CSF1PO	11-12	11-12	10-12	10-12	10-12	10-13	10-10	10-10	10-12	11-12
TPOX	8-12	8-8	8-9	8-8	11-11	8-10	11-11	8-8	11-11	8-11
AMEL	X-X	X-X	X-X	X-Y	X-X	X-Y	X-X	X-Y	X-X	X-Y
D8S1179	13-14	10-14	14-15	13-13	10-14	13-14	11-14	13-16	12-14	11-14
D21S11	29-31.2	28-30	28-29	28-29	29-33.2	31.2-33.2	31-31.2	29-30	29-32.2	30-30
DYS391	/	/	/	10	/	9	/	10	/	10
D2S441	11-11.3	11-11	11-11	10-11	11-15	13-14	10-14	10-11	12-15	11-14
D19S433	11-14	15-15.2	13-14.2	13-13	13-16	14-14	15-15	13-14	15-15.2	14-14
TH01	6-9.3	6-10	7-7	6-9	9-9.3	9-9.3	9.3-9.3	6-6	9-9.3	8-9.3
FGA	22-25	21-26	20-22	20-23	23-26	23-23	23-24	20-22	20-21	22-23
D22S1045	11-15	16-16	16-16	15-15	16-16	11-16	16-16	15-17	16-16	15-16
D5S818	12-13	12-13	11-13	9-13	12-12	9-10	9-12	10-13	12-13	11-12
D13S317	8-10	12-12	11-12	11-13	11-11	12-13	8-13	8-11	11-12	9-14
D7S820	9-10	7-11	10-11	12-12	11-11	8-8	8-8	9-11	10-11	12-12
D10S1248	14-14	14-16	15-16	13-15	14-14	14-15	14-14	13-14	15-16	14-14
D1S1656	15-15	15-15	15-16	12-17.3	13-17.3	12-15	16.3-17.3	15-17.3	17.3-18	11-17
D12S391	22-23	23-23	20-26	17-23	18.3-19	18.3-23	16-18	17.3-22	18-20	18.3-20
D2S1338	17-19	17-23	17-17	17-21	17-23	17-24	17-24	17-25	17-24	19-23
YINDEL	/	/	/	2	/	2	/	2	/	2
D18S51	13-14	12-13	12-17	12-13	12-14	14-20	13-14	13-13	10-12	14-16

Table S7. Genotyping results for the 23 loci by mean of NGS methodology regarding samples from buccal swabs. NGS: Next Generation Sequencing.

NGS Global Filer Single DNA Profiles (Buccal Swabs)										
Sample / Locus	19-135	19-141	19-207	19-205	19-142	19-206	19-131	19-133	19-134	18-431
D3S1358	18-18	18-18	16-16	16-18	15-17	14-16	14-15	14-17	16-18	15-18
VWA	15-18	16-17	14-17	17-17	14-17	14-17	16-17	14-18	16-18	18-18
D16S539	13-13	9-11	9-13	9-11	11-12	9-12	12-13	10-11	11-12	9-11
CSF1PO	11-12	11-12	10-12	10-12	10-12	10-13	10-10	10-10	10-12	11-12
TPOX	8-12	8-8	8-9	8-8	11-11	8-10	11-11	8-8	11-11	8-11
AMEL	X-X	X-X	X-X	X-Y	X-X	X-Y	X-X	X-Y	X-X	X-Y
D8S1179	13-14	10-14	14-15	13-13	10-14	13-14	11-14	13-16	12-14	11-14
D21S11	29-31.2	28-30	28-29	28-29	29-33.2	31.2-33.2	31-31.2	29-30	29-32.2	30-30
DYS391	/	/	/	10	/	9	/	10	/	10
D2S441	11-11.3	11-11	11-11	10-11	11-15	13-14	10-14	10-11	12-15	11-14
D19S433	11-14	15-15.2	13-14.2	13-13	13-16	14-14	15-15	13-14	15-15.2	14-14
TH01	6-9.3	6-10	7-7	6-9	9-9.3	9-9.3	9.3-9.3	6-6	9-9.3	8-9.3
FGA	22-25	21-26	20-22	20-23	23-26	23-23	23-24	20-22	20-21	22-23
D22S1045	11-15	16-16	16-16	15-15	16-16	11-16	16-16	15-17	16-16	15-16
D5S818	12-13	12-13	11-13	9-13	12-12	9-10	9-12	10-13	12-13	11-12
D13S317	8-10	12-12	11-12	11-13	11-11	12-13	8-13	8-11	11-12	9-14
D7S820	9-10	7-11	10-11	12-12	11-11	8-8	8-8	9-11	10-11	12-12
D10S1248	14-14	14-16	15-16	13-15	14-14	14-15	14-14	13-14	15-16	14-14
D1S1656	15-15	15-15	15-16	12-17.3	13-17.3	12-15	16.3-17.3	15-17.3	17.3-18	11-17
D12S391	22-23	23-23	20-26	17-23	18.3-19	18.3-23	16-18	17.3-22	18-20	18.3-20
D2S1338	17-19	17-23	17-17	17-21	17-23	17-24	17-24	17-25	17-24	19-23
YINDEL	/	/	/	2	/	2	/	2	/	2
D18S51	13-14	12-13	12-17	12-13	12-14	14-20	13-14	13-13	10-12	14-16

Table S8. Genotyping results for the 23 loci by mean of CE methodology regarding samples from urine. CE: Capillary Electrophoresis.

CE Global Filer Single DNA Profiles (Urine)										
Sample / Locus	19-149	19-212	19-210	19-208	19-211	19-209	19-145	19-147	19-148	19-154
D3S1358	18-18	18-18	16-16	16-18	15-17	14-16	14-15	14-17	16-18	15-18
VWA	15-18	16-17	14-17	17-17	14-17	14-17	16-17	14-18	16-18	18-18
D16S539	13-13	9-11	9-13	9-11	11-12	9-12	12-13	10-11	11-12	9-11
CSF1PO	11-12	11-12	10-12	10-12	10-12	10-13	10-10	10-10	10-12	11-12
TPOX	8-12	8-8	8-9	8-8	11-11	8-10	11-11	8-8	11-11	8-11
AMEL	X-X	X-X	X-X	X-Y	X-X	X-Y	X-X	X-Y	X-X	X-Y
D8S1179	13-14	10-14	14-15	13-13	10-14	13-14	11-14	13-16	12-14	11-14
D21S11	29-31.2	28-30	28-29	28-29	29-33.2	31.2-33.2	31-31.2	29-30	29-32.2	30-30
DYS391	/	/	/	10	/	9	/	10	/	10
D2S441	11-11.3	11-11	11-11	10-11	11-15	13-14	10-14	10-11	12-15	11-14
D19S433	11-14	15-15.2	13-14.2	13-13	13-16	14-14	15-15	13-14	15-15.2	14-14
TH01	6-9.3	6-10	7-7	6-9	9-9.3	9-9.3	9.3-9.3	6-6	9-9.3	8-9.3
FGA	22-25	21-26	20-22	20-23	23-26	23-23	23-24	20-22	20-21	22-23
D22S1045	11-15	16-16	16-16	15-15	16-16	11-16	16-16	15-17	16-16	15-16
D5S818	12-13	12-13	11-13	9-13	12-12	9-10	9-12	10-13	12-13	11-12
D13S317	8-10	12-12	11-12	11-13	11-11	12-13	8-13	8-11	11-12	9-14
D7S820	9-10	7-11	10-11	12-12	11-11	8-8	8-8	9-11	10-11	12-12
D10S1248	14-14	14-16	15-16	13-15	14-14	14-15	14-14	13-14	15-16	14-14
D1S1656	15-15	15-15	15-16	12-17.3	13-17.3	12-15	16.3-17.3	15-17.3	17.3-18	11-17
D12S391	22-23	23-23	20-26	17-23	18.3-19	18.3-23	16-18	17.3-22	18-20	18.3-20
D2S1338	17-19	17-23	17-17	17-21	17-23	17-24	17-24	17-25	17-24	19-23
YINDEL	/	/	/	2	/	2	/	2	/	2
D18S51	13-14	12-13	12-17	12-13	12-14	14-20	13-14	13-13	10-12	14-16

Table S9. Genotyping results for the 23 loci by mean of NGS methodology regarding samples from urine. NGS: Next Generation Sequencing

NGS Global Filer Single DNA Profiles (Urine)										
Sample / Locus	19-149	19-212	19-210	19-208	19-211	19-209	19-145	19-147	19-148	19-154
D3S1358	18-18	18-18	16-16	16-18	15-17	14-16	14-15	14-17	16-18	15-18
VWA	15-18	16-17	14-17	17-17	14-17	14-17	16-17	14-18	16-18	18-18
D16S539	13-13	9-11	9-13	9-11	11-12	9-12	12-13	10-11	11-12	9-11
CSF1PO	11-12	11-12	10-12	10-12	10-12	10-13	10-10	10-10	10-12	11-12
TPOX	8-12	8-8	8-9	8-8	11-11	6-10	11-11	8-8	11-11	8-11
AMEL	X-X	X-X	X-X	X-Y	X-X	X-Y	X-X	X-Y	X-X	X-Y
D8S1179	13-14	10-14	14-15	13-13	10-14	13-14	11-14	13-16	12-14	11-14
D21S11	29-31.2	28-30	28-29	28-29	29-33.2	31.2-33.2	31-31.2	29-30	29-32.2	30-30
DYS391	/	/	/	10	/	9	/	10	/	10
D2S441	11-11.3	11-11	11-11	10-11	11-15	13-14	10-14	10-11	12-15	11-14
D19S433	11-14	15-15.2	13-14.2	13-13	13-16	14-14	15-15	13-14	15-15.2	14-14
TH01	6-9.3	6-10	7-7	6-9	9-9.3	9-9.3	9.3-9.3	6-6	9-9.3	8-9.3
FGA	22-25	21-26	20-22	20-23	23-26	23-23	23-24	20-22	20-21	22-23
D22S1045	11-15	16-16	16-16	15-15	16-16	11-16	16-16	15-17	16-16	15-16
D5S818	12-13	12-13	11-13	9-13	12-12	9-10	9-12	10-13	12-13	11-12
D13S317	8-10	12-12	11-12	11-13	11-11	12-13	8-13	8-11	11-12	9-14
D7S820	9-10	7-11	10-11	12-12	11-11	8-9	8-8	9-11	10-11	12-12
D10S1248	14-14	14-16	15-16	13-15	14-14	14-15	14-14	13-14	15-16	14-14
D1S1656	15-15	15-15	15-16	12-17.3	13-17.3	12-15	16.3-17.3	15-17.3	17.3-18	11-17
D12S391	22-23	23-23	20-26	17-23	18.3-19	18.3-23	16-18	17.3-22	18-20	18.3-20
D2S1338	17-19	17-23	17-17	17-21	17-23	17-24	17-24	17-25	17-24	19-23
YINDEL	/	/	/	2	/	2	/	2	/	2
D18S51	13-14	12-13	12-17	12-13	12-14	14-20	13-14	13-13	10-12	14-16

Table S10. The mixture samples DNA concentration.

Sample Code	[] DNA
Major Contributor (19-133)	55,911 ng/µL
Minor contributor (19-205)	108,248 ng/µL
TB-1-2	53,355 ng/µL
TB-1-4	51,619 ng/µL
TB-1-6	50,266 ng/µL
TB-1-8	49,572 ng/µL
TB-1-10	47,569 ng/µL
TB-1-20	44,894 ng/µL
Major Contributor (19-147)	13,26 ng/µL
Minor contributor (19-208)	0,687 ng/µL
UR-1-2	12,231 ng/µL
UR-1-4	10,851ng/µL
UR-1-6	9,429 ng/µL
UR-1-8	7,144 ng/µL
UR_1:10	6,010 ng/µL
UR-1-20	4,987 ng/µL
